# Regulation of eosinophil recruitment and heterogeneity during allergic airway inflammation

**DOI:** 10.3389/falgy.2025.1585142

**Published:** 2025-04-10

**Authors:** Lisa-Marie Graf, Daniel Radtke, David Voehringer

**Affiliations:** Department of Infection Biology, University Hospital Erlangen and Friedrich-Alexander University Erlangen-Nuremberg (FAU), Erlangen, Germany

**Keywords:** eosinophils, allergic airway inflammation, type 2 immunity, asthma, IL-5

## Abstract

Eosinophils represent a granulocyte cell type that is strongly associated with type 2 inflammatory conditions. During steady state conditions few eosinophils are found in lung tissue, though they may contribute to homeostasis. In allergic airway inflammation, eosinophils are strongly increased and associated to disease severity. The underlying type 2 immune response tightly regulates eosinophil development, recruitment, survival, and heterogeneity. Inflammatory eosinophils in the lung are unfavourable, as they can cause tissue damage, amplify type 2 immunity and induce bronchial obstruction by expelling granular proteins and cytokines. In this review we provide an overview about mechanisms regulating development of eosinophils in the bone marrow and their extravasation into the lung including recent findings on induction and diversity of eosinophilia in allergic airway inflammation.

## Introduction

Eosinophils were firstly described in 1879 by Paul Ehrlich, who named them “eosinophils” because they appeared as granulated cells with an affinity for eosin staining (1). The granules of eosinophils contain various potent defensive proteins like major basic protein (MBP, *PRG2*), eosinophil cationic protein (ECP, *RNASE3*), eosinophil peroxidase (EPO, *EPX*), and eosinophil-derived neurotoxin (EDN, *RNASE2*), which can be toxic to helminths, viruses and bacteria, but can also damage host cells (2). Eosinophils are highly conserved cells across many vertebrate species (3). Human and mouse eosinophils both have been well characterized and they share various similarities but also harbour differences. One example are surface markers, which are frequently used to define eosinophils in flow cytometric or immunohistologic analyses. They share the expression of CCR3 and CD11b (*ITGAM*, human and mouse), show expression of the functional paralogues SIGLEC-8 (human) and SIGLEC-F (mouse) and express the orthologue proteins EMR1 (*ADGRE1*, human) and F4/80 (*Adgre1*, mouse). They differ in the expression of the IgE-receptor FcεRI, which is only present on human eosinophils (4, 5). Murine and human eosinophils are comparable in size and occur in a similar frequency in the blood, however, human eosinophils display greater granularity (6).

In healthy individuals, eosinophils make up less than 5% of leukocytes in peripheral blood and occur in the lung only in low numbers. They are short lived as they survive only about 8–12 h in blood circulation and up to 12 days in some tissues (7). Despite the low frequency of eosinophils in the homeostatic lung, eosinophils were shown to play a role in lung homeostasis. As such, they were described to inhibit the maturation of dendritic cells (DCs) and the type 2 helper T-cell (Th2) response in allergy (8). In eosinophil-associated diseases eosinophil counts in the blood increase, also termed as eosinophilia. Eosinophilia can occur especially during parasitic infections, in allergic diseases and certain autoimmune diseases (9). The incidence of allergic diseases and asthma is increasing worldwide and constitutes a major health concern (10). Depending on the eosinophil counts in blood, allergic asthma is classified either as eosinophilic or non-eosinophilic. The latter is rather characterized by the absence of blood ILC2s and a mixed granulocytic or paucigranulocytic phenotype including neutrophils (11). In eosinophilic asthma, eosinophil counts in the blood of asthma patients correlated with severity of asthma and exacerbation rates (12). Moreover, eosinophilic asthma can be subdivided into atopic (allergic) and non-atopic (non-allergic) asthma. Atopic asthma is classified by the contribution of type 2T-helper cells (Th2) cells, whereas in non-atopic asthma primarily type 2 innate lymphoid cells (ILC2) are responsible for eosinophilia (13). The determination of such asthma endotypes is crucial for selection of the appropriate treatment options. Severe asthma is commonly treated with glucocorticoids, corticosteroids, long-acting beta-adrenergic receptor agonists and leukotriene receptor antagonists. However, as eosinophils contribute to asthma severity and exacerbations, reduction of eosinophil numbers is another treatment option in eosinophilic asthma. One target of treatment is IL-5, which drives eosinophil differentiation and survival. Therefore, IL-5 neutralizing antibodies like Reslizumab and Mepolizumab and the IL-5 receptor antagonist Benralizumab are used to treat eosinophilic asthma and can reduce exacerbations by approximately 50%. As IL-4 and IL-13 cytokines are considered to be crucial for eosinophil recruitment, inhibition of these cytokines or the IL-4 receptor alpha chain is another treatment option to reduce eosinophils, although with rather inconsistent effects on exacerbation rates (14).

As eosinophils are a hallmark of eosinophilic asthma and contribute to severity of asthma, it is of great interest to understand how eosinophils develop in the bone marrow and how recruitment and migration of eosinophils to the lung is regulated.

## Eosinophil development in the bone marrow

It is long known, that eosinophils develop in the bone marrow from a CD34^+^ progenitor population. Further development from this progenitor into eosinophils is dependent on various transcription factors like c/EBPα, c/EBPε, PU.1, IRF8, Gata-1 and Gata-2 (15). Especially Gata-1 is important for eosinophil development, as impaired expression of Gata-1 in the ΔdblGATA mouse strain selectively depletes all eosinophils (16) and constitutive expression of Gata-1 in human progenitors enforces eosinophil fate (17). Drissen et al. (18) propose a new model of haematopoiesis in which a megakaryocyte and an eosinophil-mast cell progenitor (EoMP) population arise separately from the macrophage/neutrophil lineage in a Gata-1-dependent manner. This EoMP gives rise to eosinophils, mast cells, and basophils (18). The lineage commitment between basophils and eosinophils depends on the sequence of transcription factor activation. c/EBPα expression prior to Gata-2 seems to commit progenitors to become eosinophils, the reverse sequence of expression rather induces basophils (19). c/EBPα is essential in eosinophil development as it binds to promoters of eosinophil-determining genes and inhibits friend of *Gata* (*Fog*) expression, which at some point inhibits expression of eosinophil-determining genes (20). Recently, Jorssen et al. confirmed the aforementioned Gata-1 dependent lineage fate by using combined scRNA sequencing and transcriptomic analysis (21). Their approach also identified four distinct stages of eosinophil development. One important step in eosinophil development is the upregulation of the chemokine receptor CCR3, which is critical for chemotaxis of eosinophils towards inflamed tissues (22).

IL-5 is a prominent cytokine in eosinophil development and IL-5 overexpressing mice show a massive eosinophilia (23). In line, the IL-5 receptor alpha chain (also named CD125) is expressed by early eosinophil progenitors (21, 23). However, IL-5 is not essential for basal eosinophil development as IL-5 blocking antibodies reduce eosinophil counts in eosinophilic diseases drastically, but not completely (24). Further, IL-5 knock-out (k.o.) mice still harbour a low number of eosinophils in homoeostasis (25). The mechanism of how eosinophils develop in the absence of IL-5 still needs to be investigated. The IL-5, IL-3 and GM-CSF receptors share the common beta chain (βc), which is known to activate various signalling pathways, including the MAPK pathway by a βc-Shc-Grb2 cascade (26). Our group showed that Grb2 plays an important role for eosinophil development (27). Moreover, it was reported that Grb2 binds to the phosphatase PTPN11 (SHP-2) in IL-5 stimulated eosinophils (28). SHP-2, which also can be induced by IL-3, IL-5, and GM-CSF, was described as essential factor for eosinophil bone marrow development and as an inducer of c/EBPα expression (29–31). These studies provide hints on how cytokines might regulate eosinophil development. However, a direct link between receptor signalling and eosinophil-lineage inducing transcription factors is missing.

Eosinophil development was further shown to depend on IL-33. IL-33 is a member of the IL-1 cytokine family and signalling is mediated via the IL-33 receptor ST2 (*IL1RL1*) (32). IL-33 is mainly produced by epithelial cells and released in case of epithelial damage, which is also occurring in allergic airway inflammation and mainly stimulates ILC2s and Th2 cells. In mice that lack IL-33 or the Il-33 receptor ST2, eosinophils could not develop properly, and eosinophil numbers in blood and periphery were strongly reduced (33). Mature eosinophils were shown to be ST2 positive, but whether eosinophil precursors in the bone marrow express ST2 on the surface is controversial in literature. Whereas Tsuzuki et al. found ST2 expression on eosinophil progenitors (34), other data could not confirm this finding (33). Interestingly, Drissen et al. found *Il1rl1* (the gene encoding ST2) expression in Gata1-EGFP positive progenitors, however, they could not confirm ST2 expression on the surface of the respective cells (18). This raises the question whether the effect of IL-33 on eosinophil development is direct or indirect via other cells responsive to IL-33. Our group has performed analysis on mixed bone marrow chimeras, reconstituted with a 50:50 mix of *Il1rl1*^−/−^ and WT bone marrow. In this setup, *Il1rl1*^−/−^ and WT eosinophils share the same environment so that alterations in the ratio of *Il1rl1*^−/−^ and WT eosinophils indicate cell-intrinsic effects of *Il1rl1* deficiency. As *Il1rl1^−/−^* eosinophils accumulated in the lung with similar numbers as WT eosinophils after allergen challenge, the effect of IL-33 on eosinophil development appears to be non-intrinsic and rather indirect most likely by activation of ILC2s (35).

Despite substantial knowledge about transcription factor regulation in eosinophils development, it is not fully elucidated how extracellular factors induce the shift toward eosinophil commitment.

## Chemotactic migration of eosinophils towards the inflamed lung

**Figure 1:**
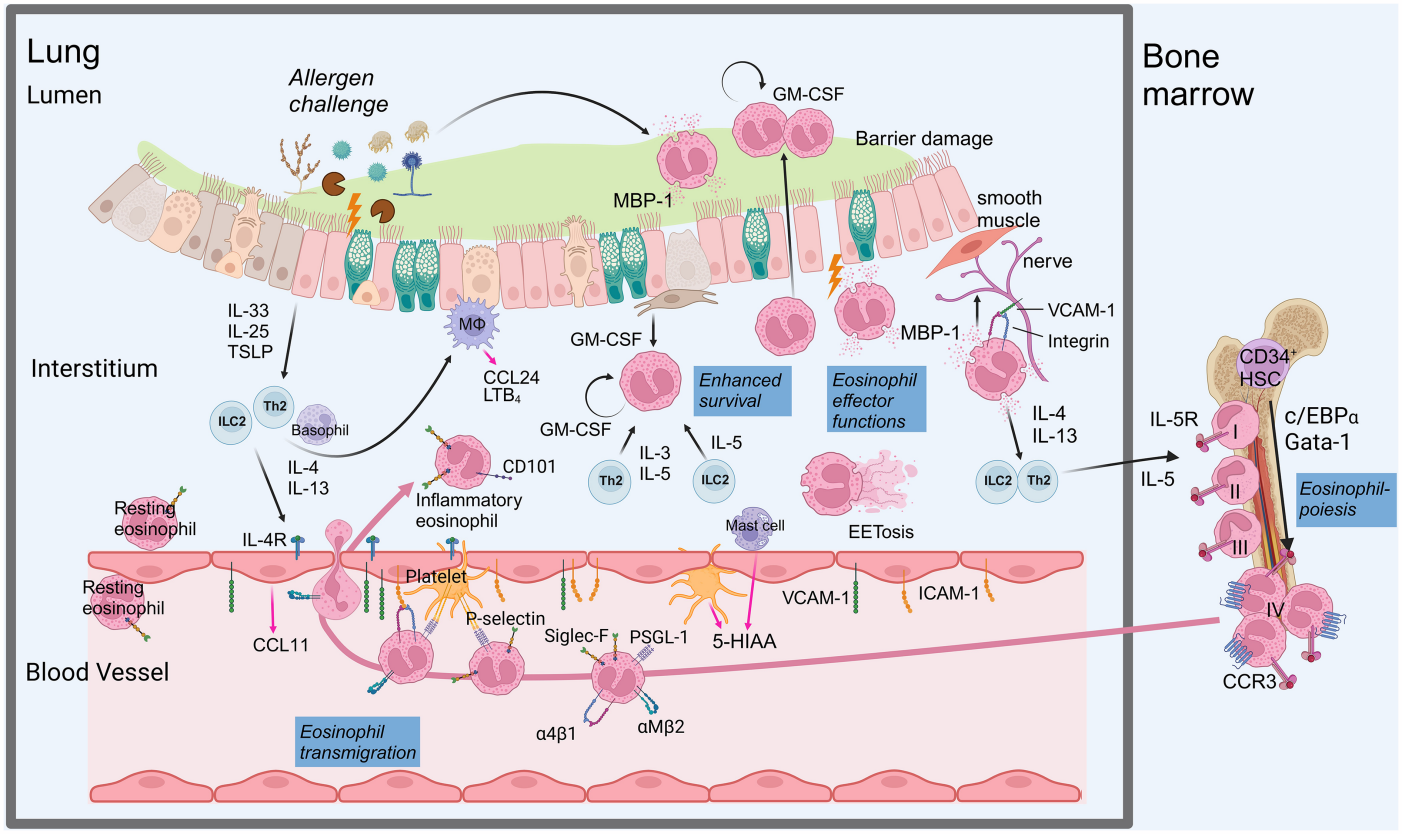
Regulation of allergen-induced lung eosinophilia. Allergens that reach the lung can damage the epithelium, leading to the secretion of alarmins, which then elicit a type 2 immune response. Type 2 cytokines induce eosinophil-chemotactic factors (pink arrows) and prime the endothelium to allow eosinophil transmigration. Thus, inflammatory eosinophils are recruited to the lung, undergo adhesion, rolling, and transmigration through the endothelial layer. In the lung tissue eosinophil survival is enhanced by paracrine and autocrine GM-CSF as well as T cell-derived IL-3 or IL-5 from Th2 cells or ILC2s. Inflammatory eosinophils execute various functions, like damaging the epithelium, activating nerves and inducing further activation of type 2 immune cells. Inflammatory factors, released by lung cells feedback to the bone marrow to increase eosinophil development, mainly driven by IL-5. Eosinophil development in the bone marrow can be divided into four stages and depends on combined action of transcription factors. Created in BioRender. Pollock, J. (2025) https://BioRender.com/m48o078.

After eosinophils have matured in the bone marrow, they will enter the blood circulation and stay there for 8–12 h. In case of airway inflammation, chemokines are released, which attract eosinophils to the lung (Figure 1). The major chemokines for eosinophil attraction are eotaxin-1 (CCL11), -2 (CCL24), and -3 (CCL26), which primarily interact with the C-C-chemokine receptor 3 (CCR3). Hence, CCR3-deficient mice show reduced eosinophil infiltration into allergen-challenged lungs, in line with a prominent role of CCR3 for eosinophil attraction to the lung tissue (36). A comparison of ovalbumin (OVA)-induced asthma in eotaxin-deficient mice showed that CCL24 seems to have a greater impact on eosinophil recruitment into the bronchoalveolar lavage (BAL) than CCL11 (37). Besides CCL11 and CCL24, CCL26 was described as a third eotaxin, which can be induced by IL-4 in an endothelial cell line (38). CCL26 could also be detected in asthmatic patients and was even suggested to be more effective in eosinophil recruitment as compared to CCL11 and CCL24 (39, 40). A further parameter for regulation of immune cell migration is protein modification by sialylation, which affects various chemokine-chemokine receptor interactions. Recently it was shown, that sialylation by the sialyltransferase ST3Gal-IV is necessary for binding of CCL11 to CCR3. Therefore, ST3Gal-IV knockout mice show decreased BAL eosinophils upon lung allergen challenge (41). Whether sialylation is also necessary for binding of CCL24 and CCL26 to CCR3 is currently unknown.

Moreover, besides eotaxins, CCR3 can bind CCL5, CCL7, CCL13, and CCL15 (42, 43). Of these, CCL7 was suggested to be relevant for eosinophil recruitment to the lung in type 2 immunity (44, 45). Additionally, CCL5 and CCL13 were suggested to induce eosinophil migration (46, 47). Whether these effects are mediated via CCR3 on eosinophils or other chemokine-receptors still has to be determined.

Because knock-out of CCR3 does not fully blunt eosinophil lung recruitment in mouse studies, it seems that CCR3-independent mechanisms for eosinophil recruitment exist. Indeed, several studies suggested that eosinophils could express other chemokine receptors like CCR1, CCR4, and CXCR4 (48, 49). However, the latter seemed to have no function in eosinophil migration to inflammatory sites (50). CCR1 is important for eosinophils to be responsive to the ligand CCL3 (Macrophage inflammatory protein 1a) (49). Moreover, by using CCL6-deficient mice, it was suggested that in asthmatic mice eosinophil-derived CCL6 can bind to CCR1 and thus induces chemotaxis of eosinophils (51). Another study suggests, that dendritic cell derived CCL17 and CCL22 drives early eosinophil recruitment via eosinophil-expressed CCR4 (52).

Beside chemokines, other factors have been investigated in the course of eosinophil chemotaxis. Prostaglandin D2 induced shape changes, which seem to mobilize eosinophils from the bone marrow and prime them for chemotaxis (53, 54). Interestingly eosinophils migrated towards nematodes in a leukotriene B4 (LTB_4_)-dependent manner, which is rather known as a chemo-attractive factor for neutrophils (55, 56). Of note, LTB_4_ was also shown to be increased in asthmatic individuals (57). Therefore, there might be a link between LTB_4_ and eosinophil chemotaxis in asthma. Moreover, it was shown that mast cells and platelets can be a source for the serotonin metabolite 5-hydroxyindoleacetic acid (5-HIAA), which induces eosinophil-recruitment via the G-protein coupled receptor (GPCR) GPR35 into *Cryptococcus neoformans* infected lungs (58).

While different factors are long known in eosinophil chemotaxis to the inflamed lung, a comprehensive understanding of their combined action *in vivo* is necessary to envision future treatment options that are precise and safe.

## Transmigration of eosinophils across endothelial cells

After eosinophils got attracted to the lung, they cross the lung endothelial cell layer to enter the lung tissue. Therefore, eosinophils undergo rolling, adhesion and subsequently transmigration through the endothelial layer.

Interaction of P-selectin glycoprotein ligand-1 (PSGL-1) on eosinophils with P-selectin seems to be important for the transmigration process. P-selectin is expressed on endothelial cells, but also on platelets. Interestingly, platelets were reported to be recruited to the endothelium in murine allergic asthma models and platelets are also found in the lung of asthmatic patients (59). Platelet-deficient mice showed reduced eosinophilia in an allergic model and it is proposed that the missing PSGL-1-P-selectin interaction is responsible for this phenotype (60). Other studies suggested that the P-selectin mediated interaction of platelets with eosinophils, upregulates and activates integrin β_1_ on eosinophils, which might be relevant for transmigration (61, 62). Moreover, as mentioned before platelets were described as source for 5-HIAA, which promoted eosinophil attraction to lung tissue (58). This indicates that platelets in proximity to endothelial cells play a major role for eosinophil transmigration.

Furthermore, attachment to endothelial cells is important in eosinophil transmigration. Whether eosinophils attach also to P-selectin on endothelial cells *in vivo* is not firmly proven, as administration of anti-P-selectin antibodies blocked P-selectin on both endothelial cells and platelets. However, endothelial cells express additional selective adhesion proteins, like VCAM-1 and ICAM-1, which are important for eosinophil attachment via integrin heterodimer interaction. Eosinophils express the integrin heterodimers α_4_β_1_ (VLA-1) and α_4_β_7_, which can interact with VCAM-1 as well as the integrin dimer α_M_β_2_ which is able to interact with ICAM-1 (63). Blocking of different integrins expressed on eosinophils, suggested a prominent role for integrin α_4_ for eosinophil adhesion (64). Integrin α_4_β_7_ also interacts with MAdCAM-1 (65), but there is no evidence whether this is necessary for eosinophil transmigration to lung tissue. *In vitro* data suggests that eosinophils role more efficiently on VCAM-1- as compared to MAdCAM-1-expressing endothelial cells (66). Noteworthy, airway eosinophils from allergen-challenged patients displayed increased integrin α_M_β_2_ expression and enhanced attachment to VCAM-1 and ICAM-1 (67).

VCAM-1 expression on endothelial cells is mainly induced by TNF-α and IL-1β. However, the type 2 immune cytokines, IL-4 and IL-13 were also shown to affect VCAM-1 expression on endothelial cells. One study suggested that IL-4 induced STAT6 can bind to the *Vcam-1* promotor (68). In the skin, basophil-derived IL-4 could upregulate VCAM-1 on endothelial cells, which was necessary for eosinophil transmigration (69). Similar results were observed in house dust mite (HDM)-challenged mice, were basophil-deficient animals did not show increased levels of VCAM-1 (70). These studies indicate that VCAM-1 expression is enhanced by type 2 cytokines and facilitates eosinophil recruitment to the lung.

After attachment to adhesion molecules, eosinophils must actively migrate through the endothelial cell layer. A recent study showed that IL-13 downregulates miR-1 in endothelial cells, thereby activating various eosinophil trafficking genes (71). One of them is thrombopoietin (*Mgl*), which seems to alter P-selectin on platelets and might support eosinophil transmigration as mentioned before (72). The serotonin precursor 5-HTP was found to inhibit eosinophil transmigration via binding to the respective receptor in endothelial cells *in vitro*. Nucleotide polymorphisms in the gene encoding this receptor were found to be associated with asthma (73, 74). However, the mechanism how this receptor-activation inhibits eosinophil transmigration was not investigated so far.

Besides some basic understanding of how eosinophils attach to endothelial cells, their trans-endothelial migration is hardly studied. In contrast, the transmigration process of neutrophils is better understood and the integrin heterodimer α_M_β_2_ appears to play a crucial role in this process (75). Eosinophils also express this dimer and a similar function in eosinophils is further suggested by β_2_-deficient mice that show reduced eosinophil migration to lung tissue (76). Additionally, it was suggested that this integrin mediates migration of eosinophils via binding to periostin, an extracellular matrix protein expressed by epithelial and endothelial cells, and secreted by IL-4- and IL-13-stimulated fibroblasts (77–79).

Overall, these studies indicate that rolling, adhesion and actual transmigration of eosinophils in the lung is tightly regulated and enhanced by type 2 immune responses.

## Effects of type 2 immune cells on pulmonary eosinophilia

The type 2 immune cytokines IL-4 and IL-13 are essential for eosinophil recruitment to the allergic lung, as depletion of both cytokines abolishes eosinophilia (80). IL-4 and IL-13 are closely related and share various functions, however, also differences are observed. Whereas IL-4 can bind to the IL-4 receptor type I (IL-4RI), both cytokines can activate the IL-4RII which consists of an IL-4Rα and IL-13Rα1 subunit. Binding of IL-4/IL-13 to these receptors leads to activation of STAT6, which can induce a great variety of STAT6-dependent genes (81). In humans, STAT6 is associated with asthma and other allergic diseases, as patients with a gain of function mutation in *Stat6* showed a variety of allergic diseases, including asthma (82). In contrast, loss-of-function variants of STAT6 rather protect individuals from allergic asthma (83).

Th2 cells are main producers of IL-4 and IL-13 and were thought to be the crucial driver of eosinophilia. However, in Rag1-deficient mice which lack T and B cells or Th2-deficient mice, eosinophilia is only abrogated in some asthma models. More precisely, in Rag1-deficient mice eosinophilia is absent in models of HDM-induced asthma, allergic bronchopulmonary aspergillosis or OVA-induced asthma (84–87). In a more mechanistic approach, using mice that only lack IL-4/IL-13 in T cells, our group showed that cytokines of Th2 cells are responsible for induction of eosinophilia in OVA-induced asthma as well as in an ABPA mouse model (80, 88). Therefore, HDM-induced asthma, allergic bronchopulmonary aspergillosis or OVA-induced asthma represent Th2-dependent asthma subtypes [reviewed in (89)]. Besides Th2 cells, ILC2s are a major source of IL-13, but also produce IL-4 to a minor extent. Interestingly, mice which lack ILC2s showed reduced eosinophilia in an HDM model, despite the fact that Th2 cells are induced normally (90). Therefore, ILC2s might also contribute to eosinophilia in OVA-induced asthma or ABPA, which was not investigated. In contrast to the Th2-dependent asthma models, the models using papain extract or *Alternaria*-induced asthma still elicit eosinophilia in Rag1-deficient mice (91, 92). However, when using ILC2-deficient mice, eosinophilia is abrogated in both asthma models, indicating a major role for ILC2s in eosinophil recruitment (90). In line, in human asthma ILC2s and a strong Th2 signature are associated with eosinophilia (93, 94).

Lung epithelial cells and fibroblast express the IL-4RI and can be induced by IL-4/IL-13 signalling. Indeed, chimeric mouse experiments demonstrated that STAT6 in non-hematopoietic cells is necessary for eotaxin induction and eosinophil recruitment (95). Recently, it was demonstrated that mainly IL-13 induces CCL11 and CCL24 in fibroblasts and monocytes, which was required for eosinophil attraction in HDM-induced murine asthma (96). In addition, murine and human endothelial cells respond to IL-4 and IL-13, which can induce CCL26 (38, 71). Beside secretion of eotoxins, epithelial cells also release IL-33 which has been shown to induce NF-κB signalling in eosinophils, thus activate them (97).

Besides the response of structural lung cells to IL-4/IL-13, macrophages are also responsive to type 2 cytokines. Especially alveolar macrophages (AMs) reside in lung tissue during homeostasis. Chlodronate-based depletion of AMs, led to increased eosinophils in lung tissue and indicates a dampening role of AMs against eosinophilia in steady state (98). However, during allergic lung inflammation macrophages get polarised into M2 macrophages, dependent on STAT6 signalling. Inhibition of this conversion reduced eosinophilia in a mouse model of allergic lung inflammation (99). Despite, stimulation of macrophages with IL-4 also directly induces CCL24 expression (100), no direct effect of macrophages on eosinophil recruitment during allergic lung inflammation could be demonstrated so far.

Basophils are another relevant source of IL-4/IL-13. Whether they contribute to eosinophilia in allergic airway inflammation is controversial in the literature. Our group has established a basophil-deficient mouse (Mcpt8Cre) line, in which OVA still induced eosinophilia in the lung (101). Other groups depleted basophils via treatment with either, anti-FcεR1α or anti-CD200R3 antibodies and observed reduced eosinophilia in murine OVA or HDM asthma models (102, 103). Recently, basophils were shown to regulate the entry of Th2 cells into the lung during HDM-induced asthma, thus eosinophilia was reduced in Mcpt8Cre mice (70). Another study, which used the papain model showed that basophils could induce cytokine production in ILC2s and thereby promoted lung eosinophilia (104). Whether basophil-derived cytokines have direct impact on eotaxin production has not been reported, but it seems that basophils can indirectly induce eosinophilia via Th2 cells and ILC2s.

It is known that various type 2 immune cells have impact on eosinophilia, however, the complex mechanism of the cascade how type 2 cytokines regulate eosinophils could enhance our understanding of asthma pathology.

## Eosinophil heterogeneity and survival in inflamed lung tissue

The function of eosinophils in the lung differs between steady state and inflammatory conditions and the phenotype of murine resident (Siglec-F^lo/int^, CD101^lo^) and inflammatory eosinophils (Siglec-F^hi^, CD101^hi^) is well defined. The resident eosinophils are established in the lung early in live, around postnatal days 3–14 (105). They show an immune regulatory and homeostatic phenotype and seem to inhibit Th2 cells (8, 106). Therefore, they are considered to play a crucial role in lung homeostasis [reviewed in (107, 108)]. Inflammatory eosinophils display higher integrin expression, which indicates an infiltrating phenotype (106, 109, 110). Notably, these eosinophils are increased in OVA- or HDM-induced asthma models and asthmatic patients correspondingly display an elevated CD101^+^ eosinophil level (111, 112). Furthermore, CD101 positive and negative eosinophils differ in their airway localisation after *Nippostrongylus brasiliensis* infection and in an HDM asthma model. CD101^−^ eosinophils were located rather in the vasculature, whereas CD101^+^ ones were primarily found in the extravascular lung space (111, 113). *In vitro*, IL-4 stimulation of mouse eosinophils strongly upregulates the expression of *Cd101* (114), suggesting a role in eosinophil activation. Siglec-F upregulation on inflammatory eosinophils might also directly modulate their behaviour as signalling via Siglec F has been associated with apoptosis, but also with context-dependent enhancement of cytokine and chemokine secretion *in vitro* (35, 115, 116). Both characteristics are also described for Siglec-8, the human paralog of Siglec-F, but *in vivo* confirmation beyond apoptosis induction is missing (35, 117). Interestingly, it was shown that the eosinophils, which migrated through an endothelial cell layer upregulated CD69 and CD35 expression (118). This indicates that infiltrating eosinophils in allergic lung inflammation not only have a distinct localisation in the lung but also display a different phenotype compared to resting eosinophils.

In steady state, there are almost no eosinophils located in the BAL fluid. However, in allergic lung inflammation, inflammatory eosinophils, but not resting eosinophils, accumulate in the BAL fluid (106). Flow cytometric analysis of eosinophils, indicated that murine BAL fluid eosinophils and inflammatory lung tissue eosinophils slightly differ in their integrin expression, whereas they show similar CD101 levels (119). Noteworthy, a similar effect on activated integrin status was also observed for human BAL eosinophils (120). Recently, it was shown that IL-33 activated ILC2s secrete lipid droplets which could recruit neutrophils, but not eosinophils, to the BAL fluid in a CXCR2-dependent manner (121). Whether eosinophils are recruited actively to the BAL fluid via a similar mechanism has not been investigated. However, loss of epithelial barrier integrity, which is a key feature of asthma, could also lead to a rather passive accumulation of eosinophils in the BAL fluid. Asthma-inducing allergens often comprise proteases, which are able to digest junction proteins important for epithelial integrity (122). Similarly, IL-13 was shown to downregulate tight junction proteins, thus also damage barrier function (123, 124). If these effects on barrier integrity are important for eosinophil accumulation in the BAL fluid has not been extensively studied.

In contrast to resting eosinophils, some inflammatory eosinophils also showed expression of Trem-1, which correlated with an enhanced apoptosis-related gene signature (119). Understanding eosinophil survival during allergic lung inflammation is important, as reduced apoptosis of eosinophils correlates with asthma severity in humans (125). Interestingly, enhanced adhesion to fibroblasts correlated with the survival of eosinophils (126). Thus, localization in lung tissue might be beneficial for their survival. However, other mechanisms have been identified which enhance eosinophil survival, especially, the factors GM-CSF, IL-3 and IL-5 (127). Our group provided evidence that these factors inhibit eosinophil apoptosis by NF-*κ*B mediated upregulation of Bcl-x_L_ (128)_._ Epithelial cells and fibroblast can be a source of GM-CSF, which reduces the eosinophil apoptosis rate *ex vivo* (129, 130). Interestingly, IL-33 can induce GM-CSF release from eosinophils, which prevented apoptosis in an autocrine manner (116). Furthermore, a recent study investigated the effect of Th2-derived extracellular vesicles and found a beneficial role of these EVs for eosinophil survival in an IL-3 dependent manner (131).

Further research in the field of tension between effector function, survival and resolution is mandatory to unravel the relevance of observed eosinophil heterogeneity.

## Contribution of eosinophils to allergic lung inflammation

Whereas lung resident eosinophils seem to supress type 2 driven immunity via dendritic cells (8), inflammatory eosinophils have a more pro-inflammatory phenotype. In patients, high eosinophilia correlates with asthma severity and depletion of eosinophils has a beneficial effect in some settings, thus it is of interest to understand the function of eosinophils in allergic lung inflammation.

Gleich and colleagues discussed in their review the biological consequences of eosinophil deficiency (132). They pointed out the fact that patients lacking eosinophils, still can develop severe asthma. However, it has been shown that the number of eosinophils correlates with asthma severity and anti-IL-5 or anti-IL-5 receptor treatment showed beneficial effects in asthma patients (12, 133, 134). Thus, it seems that eosinophils have an unfavourable role during allergic lung inflammation. Eosinophils are able to produce various granular proteins and cytokines. Especially the granule protein major basic protein 1 (MBP-1) was shown to induce damage in bronchial epithelial cells (135). Recently, two publications provided mechanistic insights on how MBP-1 affects lung epithelial cells. On the one hand, MBP-1 is suggested to induce ferroptosis in the epithelium via activation of the mTORC1 signalling pathway (136). On the other hand, MBP-1 was shown to induce formation of small pores in lipid bilayers, indicating that MBP-1 might damage the membrane of human bronchial epithelial cells (137).

Besides affecting epithelial cells, eosinophil granular proteins can also modulate nerves, which is critical for bronchoconstriction. Recently, a close proximity between eosinophils and nerve bundles was observed to be increased in lungs from patients with fatal asthma (138). It was shown that activated eosinophils can bind to VCAM-1 and ICAM-1 expressed on nerves, which additionally induced their degranulation (139). Thereby, eosinophils release MBP-1, which is an antagonist of the muscarinic M2 receptor. Inhibition of this receptor leads to increased acetylcholine release by parasympathic nerves, which induces smooth muscle contraction [reviewed in (140)]. Moreover, in mice and asthmatic humans, eosinophils correlated with increased sensory nerve density, which elevated airway hyperresponsiveness (141). These studies indicate a role for eosinophil-nerve interaction leading to bronchial obstruction in allergic asthma.

Another interesting process that was described in asthma is the release of DNA-containing eosinophil extracellular traps (EET). The ability of eosinophils to form EETs was higher in patients with severe asthma compared to non-severe asthma. The EETs derived from asthmatic patient's eosinophils, induced disconnection of epithelial cells and pro-inflammatory cytokine release by epithelial cells *in vitro* (142). Similar results could be observed in mouse experiments, where intranasal treatment of mice with EETs induced cytokine upregulation in epithelial cells. Additionally, ILC2 activation and eosinophil recruitment were induced in these mice (143). These studies indicate that EETs might contribute to the pathological role of eosinophils in asthma.

Eosinophils also produce the cytokines IL-4 and IL-13 (144–146). Experiments with eosinophil-deficient mice demonstrated that eosinophils are required for T cell recruitment into OVA-challenged lungs and high Th2 cytokine level (147). We observed similar effects in a mouse ABPA model, where eosinophils were required for a strong Th2 response, type-2 cytokine production and IgE induction (88). Besides the effect of eosinophils on Th2 cells, a recent report also describes an effect on ILC2s. Eosinophil-deficient (iPHIL) mice and mice lacking IL-4/IL-13 producing eosinophils displayed a reduced ILC2 response in HDM- and OVA-induced asthma models. Moreover, effects of eosinophils on ILC2 proliferation, recruitment and activation in an *in vitro* setting were proposed (148). These data suggest a positive feedback-loop, in which eosinophils induce Th2 cells and ILC2s, which are in turn important for eosinophil recruitment.

In contrast to the effects observed in acute inflammation, eosinophils are also discussed to contribute to resolution of inflammation. Indeed, eosinophil-deficient PHIL mice showed reduced resolution of airway inflammation, because of reduction in IL-10 producing eosinophils (149). Resolution of inflammation requires efficient removal of apoptotic cells, which would otherwise trigger further inflammation and is a mainly macrophage-dependent process. In lymph nodes, eosinophils were shown to induce CXCL13 expressing macrophages which contributed to the resolution of inflammation (150). Moreover, eosinophils themselves have been reported to take up apoptotic cells which then induced an anti-inflammatory gene expression signature in eosinophils, related to wound healing responses (151).

According to these recent studies on eosinophil functions, it becomes more evident that eosinophils indeed play a major role during allergic airway inflammation.

## Conclusion

Impaired lung function can drastically impair the quality of life and increased numbers of eosinophils in the lung correlate with severity and exacerbations in asthma. Despite the availability of modern drugs that efficiently reduce eosinophilia, our understanding of eosinophil development, tissue recruitment, effector functions and survival remains incomplete and requires further investigations.

Eosinophilia in allergic lung inflammation is tightly regulated. *De novo* production of eosinophils in the bone marrow is increased via IL-5 release from Th2 cells and ILC2s. As published data indicates that eosinophilia is not completely abolished in IL-5 ko mice, further investigation on how eosinophil development is promoted by type 2 immunity is needed. From the bone marrow, eosinophils are recruited into the lung mainly in a chemotactic manner. The newly recruited eosinophils appear to have an inflammatory phenotype. Knowledge about the eosinophil transmigration process into the lung is limited and requires further investigation. In addition, it remains unclear how positioning within the tissue environment influences the effector functions of inflammatory and resting eosinophils. To further study the complex biology of different eosinophil subtypes, single-cell sequencing was used to describe distinct eosinophil subtypes in the intestine (152). This approach has not been used to study lung eosinophils so far, but could give insights into various lung eosinophil subtypes. Another study failed to gain single cell RNA-sequencing data from bone marrow eosinophils, most probably because eosinophils express large quantities of RNAses (21). Therefore, proteomic approaches might be better suited to study differences between eosinophil subtypes.

Despite the fact that many studies examined eosinophilia in allergic lung inflammation, the greater context still lacks functional understanding. Moreover, distinct eosinophil subtypes may execute specific functions in the context of type 2 immune responses. A better understanding of eosinophil subset features and functional importance is crucial to develop new therapeutic approaches to interfere with eosinophil associated pathology.
